# Vasoconstrictor Mechanisms in Chronic Hypoxia-Induced Pulmonary Hypertension: Role of Oxidant Signaling

**DOI:** 10.3390/antiox9100999

**Published:** 2020-10-15

**Authors:** Simin Yan, Thomas C. Resta, Nikki L. Jernigan

**Affiliations:** Vascular Physiology Group, Department of Cell Biology and Physiology, University of New Mexico Health Sciences Center, Albuquerque, NM 87131, USA; syan@salud.unm.edu (S.Y.); tresta@salud.unm.edu (T.C.R.)

**Keywords:** reactive oxygen species, pulmonary vasoconstriction, calcium influx, calcium sensitization, pulmonary hypertension, chronic hypoxia

## Abstract

Elevated resistance of pulmonary circulation after chronic hypoxia exposure leads to pulmonary hypertension. Contributing to this pathological process is enhanced pulmonary vasoconstriction through both calcium-dependent and calcium sensitization mechanisms. Reactive oxygen species (ROS), as a result of increased enzymatic production and/or decreased scavenging, participate in augmentation of pulmonary arterial constriction by potentiating calcium influx as well as activation of myofilament sensitization, therefore mediating the development of pulmonary hypertension. Here, we review the effects of chronic hypoxia on sources of ROS within the pulmonary vasculature including NADPH oxidases, mitochondria, uncoupled endothelial nitric oxide synthase, xanthine oxidase, monoamine oxidases and dysfunctional superoxide dismutases. We also summarize the ROS-induced functional alterations of various Ca^2+^ and K^+^ channels involved in regulating Ca^2+^ influx, and of Rho kinase that is responsible for myofilament Ca^2+^ sensitivity. A variety of antioxidants have been shown to have beneficial therapeutic effects in animal models of pulmonary hypertension, supporting the role of ROS in the development of pulmonary hypertension. A better understanding of the mechanisms by which ROS enhance vasoconstriction will be useful in evaluating the efficacy of antioxidants for the treatment of pulmonary hypertension.

## 1. Introduction

The pulmonary circulation is normally a low resistance and low-pressure system with a mean pulmonary arterial pressure (mPAP) less than 20 mmHg [1]. Pulmonary hypertension (PH) is diagnosed by resting mPAP greater than 20 mmHg accompanied by pulmonary vascular resistance ≥ 3 Wood Units in pre-capillary PH, and classified as either idiopathic (pulmonary arterial hypertension, PAH, WHO Group 1) or secondary to left heart diseases (WHO Group 2), chronic hypoxic lung diseases (WHO Group 3), thrombosis (WHO Group 4), or of unclear reasons (WHO Group 5) [1].

PH is typically not diagnosed at early stages until the appearance of heart failure symptoms [2] such as dyspnea, palpitation and lower-extremity edema, which can ultimately lead to morbidity and mortality. This review will address many forms of PH with a focus on chronic hypoxia (CH)-induced PH, which occurs in patients with chronic obstructive pulmonary diseases (COPD), restrictive lung diseases, sleep apnea and in residents at high altitude. 

**Figure 1:**
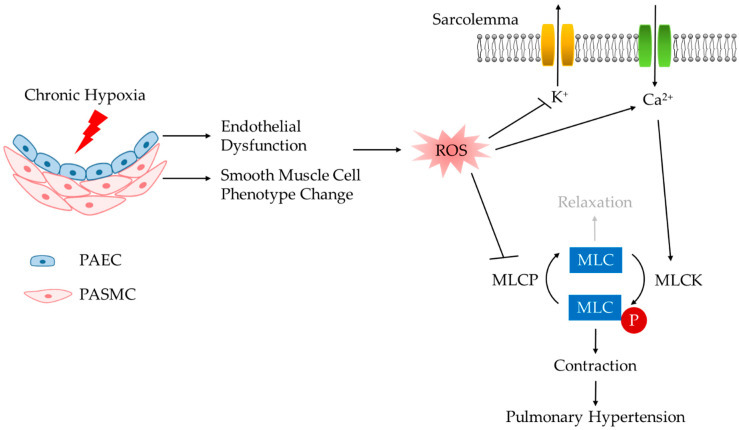
Enhanced vasoconstriction resulting from chronic hypoxia-induced functional alterations of endothelial and smooth muscle cells contributes to pulmonary hypertension. PAEC, pulmonary arterial endothelial cell; PASMC, pulmonary arterial smooth muscle cell; ROS, reactive oxygen species; MLCK, myosin light chain kinase; MLCP, myosin light chain phosphatase; MLC, myosin light chain.

Narrowing of pulmonary arteries (PAs) as a result of both structural (pulmonary arterial remodeling) and functional changes (vasoconstriction) contributes to increased vascular resistance that is pivotal to the pathogenesis of PH. Although pulmonary arterial wall thickening is observed in CH-induced PH [3,4,5], augmented vasoconstriction, manifested as both resting pulmonary arterial tone and reactivity to endogenous vasoconstrictors, plays an indispensable role in this disease [6,7,8,9,10]. Enhanced pulmonary arterial vasoconstriction results from pulmonary arterial smooth muscle cell (PASMC) hyperreactivity mediated by cytosolic Ca^2+^-dependent and Ca^2+^-sensitization mechanisms [11,12,13,14,15,16,17,18,19,20,21,22] as well as pulmonary arterial endothelial cell (PAEC) dysfunction via unbalanced production of vasoconstrictors over vasodilators [23]. Increased reactive oxygen species (ROS) have been widely reported to mediate augmented pulmonary arterial constriction [3,12,18,19,20,22,24,25,26,27,28] (Figure 1), and antioxidation strategies provide therapeutic efficacy in animal models of PH [3,29,30,31,32,33,34,35,36,37]. 

## 2. ROS in the Pathogenesis of PH

**Figure 2:**
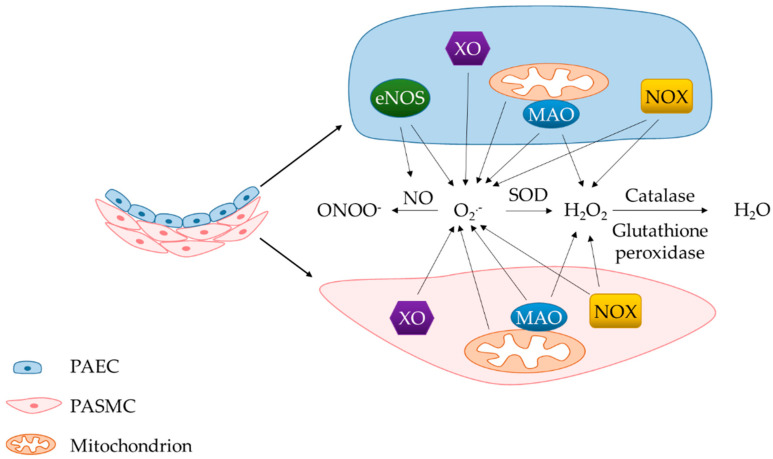
O_2_^.−^ is generated from various enzymatic sources and converted to other forms of ROS, including H_2_O_2_ and ONOO^−^. H_2_O_2_ can also be produced from NOX4 and MAOs. PAEC, pulmonary arterial endothelial cell; PASMC, pulmonary arterial smooth muscle cell; ROS, reactive oxygen species; O_2_^.−^, superoxide; H_2_O_2_, hydrogen peroxide; ONOO^−^, peroxynitrite; H_2_O, water; NOX, NADPH oxidase; eNOS, endothelial nitric oxide synthase; XO, xanthine oxidase; MAO, monoamine oxidase; SOD, superoxide dismutase.

ROS are a group of oxygen-derived molecules with one or more unpaired electrons in their outer orbit. Superoxide anions (O_2_^.−^) are formed when molecular oxygen (O_2_) receives an electron, and is derived from various sources including NADPH oxidases (NOXs), mitochondria, endothelial nitric oxide synthase (eNOS), xanthine oxidase (XO) and monoamine oxidases (MAOs). The other two forms of ROS, peroxynitrite ion (ONOO^−^) and hydrogen peroxide (H_2_O_2_), are derivatives of O_2_^.−^. Specifically, the combination of O_2_^.−^ and nitric oxide (NO) produces ONOO^−^ and partial reduction of O_2_^.−^ by superoxide dismutase (SOD) generates H_2_O_2_. There are three known SOD isoforms found in mammals including SOD1 (Cu-Zn SOD), located in the cytoplasm and intermembrane space of mitochondria, SOD2 (Mn SOD), located in the mitochondrial matrix, and SOD3 (Cu-Zn SOD), located extracellularly [38]. H_2_O_2_ is fully reduced to water (H_2_O) by catalase or glutathione peroxidase (Figure 2). 

Under normal conditions, ROS are essential signaling molecules that are tightly regulated to maintain physiological homeostasis, regulate cellular proliferation, and host defense. Within the vasculature, ROS contribute to basal endothelial cell proliferation/migration [39,40], as well as smooth muscle cell differentiation [41]. ROS can also participate in vasomotor responses such as autoregulation [42], endothelium-dependent vasodilation [43,44], flow-mediated vasodilation [45], hypoxic pulmonary vasoconstriction (HPV) [46,47] and hyperoxia-induced vasoconstriction [48,49,50]. At physiological concentrations, H_2_O_2_ elicits vasodilation in the pulmonary circulation [51,52,53,54] and diminishes HPV in CH animals [55]. In addition, oxidation of protein kinase G Iα by oxidants (H_2_O_2_, glutathione disulfide, and protein-bound persulfides) following CH counteracts enhanced PA constriction and is protective during PH development [56], suggesting that under certain conditions ROS can play a protective role. However, H_2_O_2_ has also been reported to be detrimental in PH [29,57,58]. This contradictory effect of H_2_O_2_ in the pulmonary circulation may be due to a variety of conditions, including the concentration of H_2_O_2_, experimental setup, cell type affected and intracellular signaling mechanism involved. Other forms of ROS involved in the pathogenesis of PH include O_2_^.−^ [3,12,26,29,59,60] and ONOO^−^ [61,62,63]. Although physiological levels of ROS are indispensable in maintaining vascular homeostasis, excess production leads to disease development [64,65,66,67] as detailed in the following sections.

**Figure 3:**
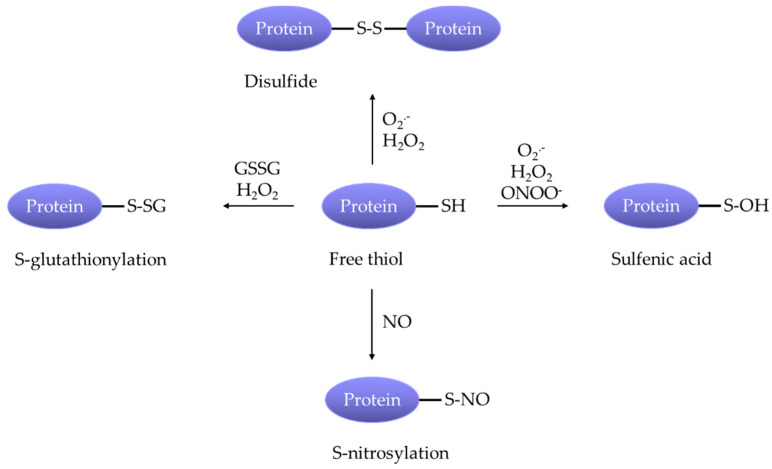
ROS facilitate pulmonary arterial constriction in pulmonary hypertension by make various posttranslational modifications at cysteine residues of ion channels and molecules.

O_2_^.−^ is considered to be highly reactive and can mediate cell signaling either directly or through its derivatives, H_2_O_2_ and ONOO^−^. These ROS participate in signaling transduction by making posttranslational modifications to ion channels, protein kinases and other signaling molecules [68,69,70]. Such protein modifications include oxidation of tyrosine, tryptophan, histidine, lysine, methionine and cysteine residues [70]. In this review, we discuss the ROS-induced functional alterations to relevant Ca^2+^ channels, K^+^ channels and other proteins that contribute to enhanced PA constriction in PH. Despite the observed effects of ROS, current knowledge about the exact chemical reactions, amino acid residues affected, and resulting protein structural changes is still limited. The most widely documented modifications in this setting are cysteine oxidative modifications (Figure 3), including S-glutathionylation (e.g., L-type voltage-gated Ca^2+^ channels [71,72] and STIM1 [73]), disulfide formation (e.g., ASIC1 [74], voltage-gated K^+^ channels [75,76] and RhoA [77]), and sulfenic acid formation (e.g., voltage-gated K^+^ channel [78]).

### 2.1. Increased ROS Production in PH

#### 2.1.1. NADPH Oxidase Family 

The NOX family consists of a group of enzymes that transfer an electron from NADPH to O_2_, therefore generating O_2_^.−^. NOX-derived ROS were first identified as effectors from phagocytes responsible for host defense [79] and later as mediators in various cellular processes. All enzymes within this family contain one of the seven ROS-generating catalytic homologs including NOX1, NOX2, NOX3, NOX4, NOX5, DUOX1 and DUOX2 [79]. Some of them are reported to be expressed within the pulmonary vasculature, as summarized in Table 1. NOX enzymes are comprised of several subunits, both catalytic and regulatory, that are located both intracellularly and extracellularly. This fact makes it possible for enzyme function to be regulated by its associated regulatory subunits as well as various intra- and extracellular signals [79,80]. 

NOX1 expression is greater in PAs from PAH patients compared to vessels from control patients [83] and contributes to the proliferation of both PAEC [83] and PASMC [86]. In monocrotaline-induced PAH model, NOX1 expression is increased in PASMCs [87]. Moreover, N-acetylcysteine, which suppresses NOX1 expression, is protective against monocrotaline-induced PAH [86]. In addition to PAH, NOX1 has also been shown to participate in PH elicited by CH as evidence by effects of genetic global deletion of NOX1 to abolish the CH-induced elevation in right ventricular systolic pressure (RVSP), right ventricle (RV) hypertrophy and PA remodeling in mice [98]. 

NOX2 expression is upregulated in response to prolonged [29] in vitro hypoxia. Using PAs isolated from wild type and gp91phox deficient mice, Liu et al. [24] discovered that NOX2-derived O_2_^.−^ production in PAs is higher after CH exposure. Additionally, augmented PA contraction to ET-1 following CH is NOX2 dependent [24]. Evidence from our group also supports that NOX2-derived ROS contribute to enhanced PA constriction following CH [18,22]. 

NOX4 is unique among the NOX family since it is intrinsically active once expressed [99,100,101]. Although biochemical evidence suggests H_2_O_2_ is the major product of NOX4 [99,100,101], NOX4-dependent generation of both O_2_^.−^ [82,97] and H_2_O_2_ [82,95,96] has been observed in the pulmonary vasculature. NOX4 expression in PAs from COPD patients [102] and CH mice [90] is higher than those from controls. NOX4 promotes proliferation of human PASMCs [90] and correlates with the severity of PA remodeling in COPD patients [102], suggesting a pathological role for NOX4 in CH-induced PH. In comparison to wild-type (WT) mice, Hood et al. [98] demonstrated that RVSP is diminished in NOX4 knockout (KO) mice following CH (10% O_2_ for 15 days). However, these NOX4 KO mice develop a similar degree of RV hypertrophy and PA remodeling as WT mice. In contrast, Veith et al. [103] reported that both global and inducible NOX4 KO mice exhibit similar elevations in RVSP after CH exposure (10% O_2_ for 21 days) as WT mice. The reason for these discrepant results is not clear, but may be due to differences in the duration of CH exposure or animal sex, as Hood et al. [98] studied only female mice. Since NOX4 does not account for monocrotaline-induced PAH [87], it appears that involvement of NOX4 in PH may differ depending on the model employed. In addition to evidence against a detrimental role of NOX4 in PH, a recent study demonstrates that increased disulfide protein kinase G Iα during CH, likely caused by NOX4-derived H_2_O_2_, opposes the pathogenesis of PH [56]. This possible beneficial effect of NOX4 in CH-induced PH is consistent with previous investigations showing the protective role of NOX4 in cardiovascular diseases. For example, endothelial NOX4 alleviates both angiotensin II-induced hypertension [104] and hemodynamic overload-induced cardiac remodeling [105]. 

#### 2.1.2. Mitochondria

Mitochondria are double-membrane organelles responsible for efficient energy generation from the electron transport chain (ETC), which is located in the inner membrane. Electrons from NADH and FADH_2_, extracted by complex I (NADH dehydrogenase) and complex II (succinate dehydrogenase) respectively, flow through the ETC and are received by O_2_ at complex IV to generate H_2_O. A small amount of ROS are inevitably produced during this process because of “electron leak” [106]. A mitochondrial antioxidation system scavenges mitochondria-derived ROS (mitoROS) so that normal cell function can be maintained. This antioxidant system is comprised of SOD2 in the matrix and SOD1 in the intermembrane space, both of which convert O_2_^.−^ into H_2_O_2_ [107,108]. H_2_O_2_ in the mitochondrial matrix is further detoxified by glutathione peroxidase 1(GPX1) [109,110] or catalase [29,110,111]. It is mainly reported that mitoROS are generated from complex I and III with O_2_^.−^ as the primary product [112]. The role of mitoROS in CH-induced PH has been studied by several groups. Human PAECs exposed to prolonged hypoxia (72 h) have greater mitoROS levels versus normoxic controls [29]. MitoROS within PAEC participate in Ca^2+^ homeostasis as supported by data that higher intracellular Ca^2+^ in PAECs from SU5416/hypoxia-induced PAH rats versus those from normoxic animals is acutely diminished by the mitochondria-targeted antioxidant MitoQ [113]. A recent study from our laboratory similarly employed the mitochondrial antioxidants MitoQ and MitoTEMPO to demonstrate that mitoROS production is greater in PASMCs from CH neonatal rats compared to normotensive animals and contributes to enhanced PA vasoconstriction following CH [114]. Since MitoQ suppresses mitochondrial O_2_^.−^ generation, it suggests the involvement of mitochondria-derived O_2_^.−^ in the pathology of PH. Using genetically modified mice, other groups reported that mitochondrial H_2_O_2_ is also pathogenic in the development of PH elicited by CH. Evidence from Adesina et al. [29] show that indices of CH-induced PH, including RVSP, RV hypertrophy and PA remodeling, are attenuated by mitochondrial catalase overexpression, which breaks down mitochondrial H_2_O_2_, but are exacerbated by SOD2 overexpression, which increases mitochondrial H_2_O_2_.

#### 2.1.3. Endothelial Nitric Oxide Synthase

NO, an important vasodilator in the pulmonary vasculature [115], is produced by eNOS using L-arginine as substrate, which requires the cofactor tetrahydrobiopterin (BH4) [116,117]. eNOS is constitutively expressed in endothelial cells and has 3 domains, a reductase domain in the C terminus, an oxygenase domain in the N terminus and a linking domain [118,119]. Binding of Ca^2+^/camodulin to the linking domain activates the enzyme, allowing NADPH oxidation to occur at the reductase domain. Flavin mononucleotide (FMN) and flavin adenine dinucleotide (FAD) of the reductase domain of one monomer pass electrons from NADPH to the heme-containing oxygenase domain of a second monomer via BH4 [117,119], which couples NADPH oxidation to L-arginine oxidation. NO is synthesized from O_2_ and L-arginine at the oxygenase domain through a multi-step chemical reaction including: (1) the combination of O_2_ with the heme group of the oxygenase domain to form a ferrous–dioxygen complex; (2) reduction of O_2_ within the ferrous-dioxygen complex to form H_2_O; and (3) oxidation of L-arginine to produce NO and L- citrulline [116,117,118,120]. Results from Vásquez-Vivar et al. [116] demonstrate that BH4 stabilizes the ferrous-dioxygen complex to prevent O_2_^.−^ generation from eNOS. Thus, without adequate availability of BH4, eNOS produces O_2_^.−^ [121]. O_2_^.−^, in turn, can oxidize BH4. BH4 oxidation is detrimental because it further reduces BH4 bioavailability and produces dihydrobiopterin (BH2) [122], a process that additionally favors O_2_^.−^ generation from eNOS [123]. Interestingly, it is also reported that increases in BH2 alone, without a change of BH4 levels, are sufficient to induce O_2_^.−^ generation [121], suggesting the BH4/BH2 ratio is key to regulation of eNOS function. Therefore, a deleterious cycle is established in which uncoupled eNOS produces O_2_^.−^, and O_2_^.−^ further uncouples eNOS.

BH4-eNOS coupling is important in maintaining physiologically low pulmonary arterial pressure as evident by the effect of BH4 deficient mice to increase endothelial O_2_^.−^ levels as well as right ventricular systolic pressure compared to wild type mice under normoxia, regardless of changes in eNOS expression [124]. In addition, pathological hypoxic exposure triggers more severe PH in BH4-deficient mice versus WT mice, which is attenuated by genetic BH4 restoration [124]. The importance of BH4-eNOS coupling in CH-induce PH is also supported by research findings from Dikalova et al. [31], in which oral BH4 administration attenuates the development of CH-induced PH in piglets. The therapeutic potential of BH4 is demonstrated by effects of BH4 treatment to reverse established CH-induced PH in rats [34]. Consistent with evidence that supplementation of BH4 [116,123,124,125,126] and an increased BH4/oxidized BH4 ratio [31,121,123] enhance eNOS activity, oral BH4 administration promotes eNOS activity, lowers lung O_2_^.−^ levels, and reverses established CH-induced PH [34]. Additionally, BH4 is shown to be beneficial in the treatment of a rat PAH model [127]. 

In addition to BH4 oxidation, O_2_^.−^ can mediate inhibition of eNOS by exogenous NO [63,128,129], a response that correlates with the clinical observation that sudden withdrawal of inhaled NO therapy worsens PH in children [130] and infants [131] with congenital heart diseases. In primary ovine PAEC cultures, Sheehy et al. [128] discovered that reduced eNOS activity by the NO donor sodium nitroprusside is partially restored by the O_2_^.−^ scavenger Tiron. Since the reduction of eNOS activity by NO is not related to cell viability, eNOS expression, subcellular localization, or phosphorylation of eNOS [128], the mechanism by which ROS mediate NO-induced eNOS inhibition remains unclear in this cell model. More in-depth mechanisms are revealed by effects of NO inhalation to inhibit eNOS activity in a lamb model [63,129]. The involvement of ROS is implicated by the fact that O_2_^.−^ and ONOO^−^ are increased in PAs following 24 h of NO inhalation [63]. The subsequent elevated nitration of eNOS by ONOO^−^ [63] is known to reduce enzyme activity [129]. Furthermore, lambs receiving polyethylene glycol-conjugated superoxide dismutase (PEG-SOD) at the same time of NO inhalation do not show rebound PH after acute NO withdrawal seen in those treated with vehicle [63]. Taken together, these data suggest exogenous NO leads to O_2_^.−^ generation in the pulmonary vasculature, which reacts with NO to produce ONOO^−^. The resultant nitration of eNOS suppresses its activity. 

#### 2.1.4. Xanthine Oxidase 

The final two biochemical reactions of purine catabolism, namely conversions of hypoxanthine to xanthine to uric acid, are catalyzed by xanthine oxidoreductase (XOR). XOR consists of one molybdenum, two different iron-sulfur centers, and one FAD that all function as electron transporters [132,133,134]. An NAD^+^-dependent form of XOR, called xanthine dehydrogenase (XDH), is constitutively expressed, which fulfills purine degradation and generates NADH [135]. However, through oxidation of cysteine residues or proteolytic cleavage, XDH can be converted into xanthine oxidase (XO) [134,136]. Due to a decrease in NAD^+^ affinity and an increase in O_2_ affinity at the FAD site, XO exhibits high xanthine/O_2_ reductase activity instead of high xanthine/NAD^+^ reductase activity seen in XDH [132,133,134,135,136]. Therefore, purine catabolism catalyzed by XO produces ROS [132,133,134,135,136]. Experimental data from Kelley et al. [137] show that XO generates both H_2_O_2_ and O_2_^.−^ with the former as the main product (>70% of ROS) under both normoxic (21% O_2_) and hypoxic conditions. Interestingly, when the O_2_ concertation is less than 10%, the proportion of H_2_O_2_ produced is inversely related to O_2_ concentration and can up to 90% in the presence of 1% O_2_ [137], indicating the involvement of H_2_O_2_ in XO-mediated diseases caused by hypoxia. 

In the context of PH, enhanced XO activity upon hypoxic exposure has been confirmed in both in vitro [138] and in vivo [35,36] studies. Experimental inhibition of XO is protective against CH-induced increases in mPAP [35], RV hypertrophy [35,36] and pulmonary arterial wall thickening [35,36] in animal models. Consistently, compared to placebo treatment, XO inhibitor treatment (allopurinol) alleviates RV hypertrophy in COPD-associated PH patients with severe airflow limitation in a double-blinded randomized controlled clinical trial [139].

#### 2.1.5. Monoamine Oxidases 

Monoamine oxidases (MAOs) catalyze the oxidative deamination of bioactive amines and are found in brain as well as various human tissues [140]. MAO type A (MAO-A) and MAO type B (MAO-B) are two identified MAO isoforms characterized by different substrate preferences [141]. In particular, MAO-A oxidizes dopamine, norepinephrine and serotonin (5-HT), while MAO-B reacts with dopamine, phenylethylamine, benzylamine and tryptamine [142,143,144,145,146]. Substrate selectivity of MAOs is determined by phenylalanine residue 208 of MAO-A and isoleucine residue 199 of MAO-B [146]. During the oxidative deamination process, FAD, a cofactor of MAOs, delivers electrons from amines to molecular O_2_ to generate ROS [147], including O_2_^.−^ [148,149] and H_2_O_2_ [150,151,152,153]. Therefore, MAOs can act as sources of ROS. One of the downstream targets of MAOs is mitochondria, which is consistent with evidence that MAOs are tethered to the outer membrane of mitochondria [154] via a C terminal transmembrane helix [155,156]. Specifically, MAOs mediate mitochondrial dysfunction [157,158,159,160] and promote mitoROS production [158,159]. Within the pulmonary circulation, MAO-A is expressed in PAs and contributes to O_2_^.−^ generation triggered by 5-HT [148]. Preliminary observations from Sun and colleagues demonstrate that expression of MAO-A is upregulated in PAH patients [161] and that the MAO-A inhibitor clorgyline partially reverses indices of PH in a PAH rat model (SU5416/hypoxia), including RVSP, RV hypertrophy and PA remodeling [161,162]. However, the role of MAOs-derived ROS in vasoconstrictor responses of PAs and their contribution to CH-induced PH remains unclear.

### 2.2. Decreased Antioxidant Capacity in PH

Augmented ROS signaling can also result from decreased antioxidant capacity. Impaired SOD activity has been reported in a variety of PH models of animals and patients [33,163,164,165,166,167,168]. Aiming at rescuing the dysfunctional SOD system, SOD mimetics have been demonstrated to alleviate indices of PH following CH [3,32,37]. As mentioned before, three SOD isoforms are found in mammals [38] and all of them are reported to be important in the pathogenesis of PH. 

#### 2.2.1. SOD1

Expression of the predominant cytosolic SOD isoform, SOD1, is lower in PAs from CH piglets [165] and CH adult rats [28] in comparison to normoxic controls, a response associated with increased O_2_^.−^ and decreased H_2_O_2_ levels. However, the role of SOD1 in CH-induced PH is not clear. Interestingly, compared to WT mice, SOD1 KO mice display elevated O_2_^.−^ levels in PAs, exhibit enhanced vasoreactivity to ET-1, as well as greater RV hypertrophy, PA remodeling and greater RVSP under normoxia [166]. Collectively, these results indicate that loss of SOD1 in response to CH may contribute to the pathogenesis of PH. 

#### 2.2.2. SOD2

SOD2 is localized to the mitochondrial matrix [38]. SOD2 expression is reported to be downregulated in PH including in a CH mouse model [169], persistent PH lamb model [170], PAH patients [168] and a fawn-hooded rat model of PAH [164]. Loss of SOD2 in PASMCs during CH exposure may promote PA remodeling since SOD2 suppresses proliferation and promotes apoptosis of hypoxic cultures of human PASMCs [171]. It is also been shown that loss of SOD2 in PAECs is involved in elevated PA constriction in PH. In a PH neonatal lamb model established by ligation of the fetal patent ductus arteriosus during late gestation, SOD2 restoration in PAECs by adenovirus vectors reduces mitochondrial O_2_^.−^ levels and restores eNOS expression [170], suggesting an improvement of PA dilation. Moreover, SOD2 transduction in PA rings from PH animals ameliorates their relaxation in response to the NO-dependent vasodilator, ATP, compared to control transduction [170]. Since the greater H_2_O_2_ production following restoration of SOD2 expression is thought to be responsible for the observed upregulation of eNOS [170], these findings suggest that mitochondria-derived H_2_O_2_ is protective. However, Adesina et al. [29] found that CH-induced RV hypertrophy, PA muscularization and increases in RSVP are exacerbated in a transgenic mouse model overexpressing SOD2 in comparison to WT mice. In this study, increased mitochondrial H_2_O_2_ is shown to be detrimental rather than protective. 

#### 2.2.3. SOD3 

SOD3 locates extracellularly by binding to extracellular matrix components such as heparan sulfate proteoglycan, collagen and fibulin-5 [38]. Since introduction of extracellular ROS by administration of XO [172] and knockdown of SOD3 by siRNA [173] in cultured human PASMCs triggers pro-proliferative and anti-apoptotic phenotypic changes, it suggests extracellular O_2_^.−^, as well as SOD3 are likely important in the pathogenesis of PH. Considering that expression [28] and activity [28,173] of SOD3 in PAs are reduced by CH exposure, genetically modified animals with SOD3 deletion have been used to study its role in CH-induced PH development. Compared to control animals, mice with smooth muscle-specific SOD3 KO [174] or global SOD3 KO [175] exhibit greater RVSP, RV hypertrophy and pulmonary arterial wall thickening following CH. In line with this, SOD3 overexpression protects against CH-induced PH [30]. In addition to favoring PASMC proliferation, extracellular ROS also participate in CH-induced extracellular matrix remodeling as SMC SOD3 deletion augments CH-induced collagen deposition in PAs [174]. While dysfunctional SOD3 has been reported to be associated with PA remodeling, the role of SOD3 in enhanced PA vasoconstriction following CH is undetermined except for evidence that SOD3 helps to maintain normal eNOS function. Nozik-Grayck and colleagues [174] found that eNOS activation and GTP cyclohydrolase-1 (GTPCH-1, a key enzyme for BH4 synthesis) levels are diminished in lungs from smooth muscle-specific SOD3 KO mice exposed to CH, while eNOS expression is unaltered. These results are consistent with the notion that the loss of SOD3 contributes to development of PH.

## 3. ROS Modulation of Augmented PA Constriction 

The effect of CH to augment PA vasoconstrictor reactivity has been convincingly demonstrated [6,7,8,9,10]. Smooth muscle contraction is triggered by an increase in intracellular Ca^2+^ levels ([Ca^2+^]_i_) via either Ca^2+^ influx or Ca^2+^ release from the sarcoplasmic reticulum (SR). Ca^2+^ binds to calmodulin and actives myosin light chain kinase (MLCK). When the regulatory light chain of myosin is phosphorylated by MLCK, cross-bridge cycling occurs and results in smooth muscle contraction (Ca^2+^-dependent mechanism). Contraction ends when phosphorylated myosin light chain is dephosphorylated by myosin light chain phosphatase (MLCP). Therefore, factors that inhibit MLCP activity can maintain smooth muscle contraction and contribute to prolonged vasoconstriction independent of changes in [Ca^2+^]_i_ (Ca^2+^ sensitization mechanism). Both increases in [Ca^2+^]_i_ in PASMCs [11,17,176,177,178,179,180] and Ca^2+^ sensitization [12,18,20,21,22,180,181,182] are known to mediate enhanced PA vasoconstriction in response to CH. 

### 3.1. ROS Modulation of Ca^2+^-Dependent Vasoconstriction 

#### 3.1.1. Ca^2+^ Influx 

**Figure 4:**
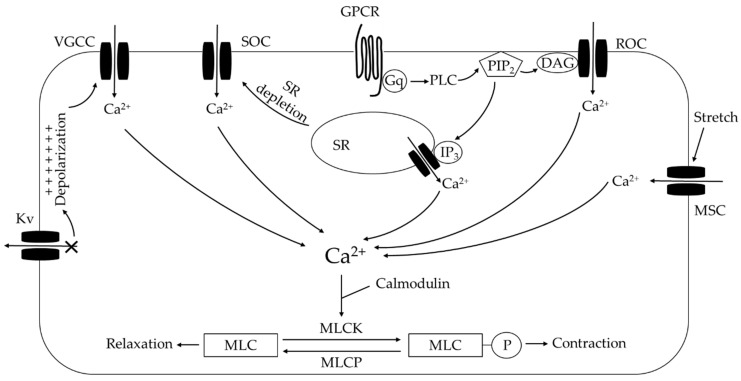
Summary of Ca^2+^-dependent influx and release mechanisms in pulmonary arterial smooth muscle cells following chronic hypoxia. See text for details. K_V_, voltage-gated K^+^ channel; VGCC, voltage-gated Ca^2+^ channel; SOC, store-operated channel; ROC, receptor-operated channel; MSC, mechanosensitive channel; GPCR, G protein-coupled receptor; PLC, phospholipase C; PIP_2_, phosphatidylinositol 4,5-bisphosphate; DAG, diacylglycerol; IP_3_, inositol triphosphate; SR, sarcoplasmic reticulum; MLCK, myosin light chain kinase; MLCP, myosin light chain phosphatase; MLC, myosin light chain.

Ca^2+^ influx is thought to contribute to the increase in [Ca^2+^]_i_ in PASMCs after CH exposure, which can involve either voltage-gated calcium channels (VGCC) or non-selective cation channels (i.e., conduct both Ca^2+^ and Na^+^) including receptor-operated channels (ROC), store-operated channels (SOC), and mechanosensitive channels (MSCs) (Figure 4). 

VGCC are gated by plasma membrane potential. Based on their sensitivity to depolarization, they are classified as high voltage-activated channels (L-, P/Q-, R-, N-type) and low voltage-activated (T-type) channels [183]. L-type and T-type VGCC are found in the pulmonary circulation [184]. Since membrane potential is a product of uneven distribution of Na^+^, K^+^ and Cl^−^ across the plasma membrane, the opening of non-selective cation channels (increased Na^+^ influx) and closing of K^+^ channels (reduced K^+^ efflux) lead to membrane depolarization and VGCC activation [185]. Plasma membrane depolarization is observed in PASMCs from CH animals [186,187] and PAH patients [188].

ROCs are controlled by diacylglycerol (DAG) generated from Gq protein-coupled receptor pathway activation [189]. SOCs are activated when intracellular SR Ca^2+^ stores are depleted [190]. When Ca^2+^ depletion in the SR is sensed by stromal interaction molecule (STIM) [190], STIM moves towards the plasma membrane and activates store-operated Ca^2+^ entry (SOCE) via Orai [191], acid-sensing ion channels (ASICs) [17,192] and transient receptor potential (TRP) channels [193]. 

Ca^2+^ channel expression and/or activity have been shown to be increased by CH and coupled to enhanced vasoconstriction (Table 2). Ca^2+^ signaling contributes to PA constriction [189]. However, it is worthwhile to note that some research findings may be animal model-specific. Previous data from our laboratory demonstrate the existence of differences in Ca^2+^ handling after CH exposure in two commonly used strains of rats [194]. In particular, CH induces an elevation in resting smooth muscle [Ca^2+^]_i_ in Wistar rats but not in Sprague-Dawley (SD) rats [194]. Additionally, SOCE is attenuated by CH in SD rat while augmented in Wistar rats [194]. 

##### Voltage-Gated Calcium Channels

**Figure 5:**
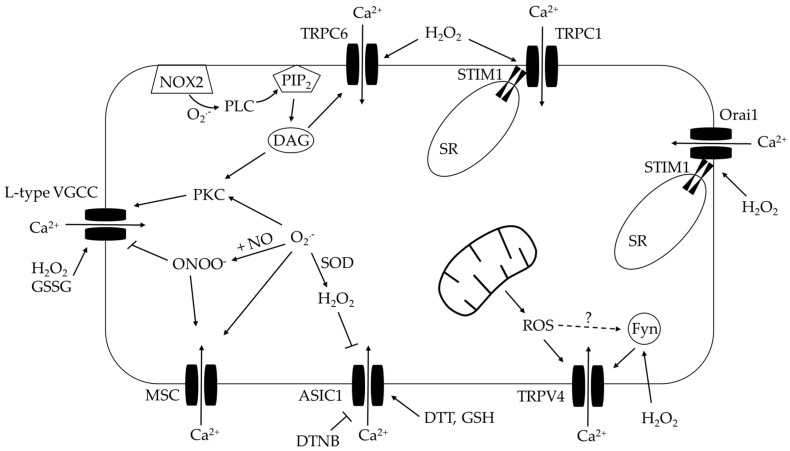
ROS modulation of Ca^2+^ influx. See text for details. L-type VGCC, L-type voltage-gated Ca^2+^ channel; TRPC, canonical transient receptor potential channel; TRPV4, transient receptor potential vanilloid 4; ASIC 1, acid sensing ion channel 1; MSC, mechanosensitive channel; NOX2, NADPH oxidase 2; PLC, phospholipase C; PIP_2_, phosphatidylinositol 4,5-bisphosphate; DAG, diacylglycerol; PKC, protein kinase C; SR, sarcoplasmic reticulum; STIM1, stromal interaction molecule 1; ROS, reactive oxygen species; O_2_^.−^, superoxide; H_2_O_2_, hydrogen peroxide; ONOO^−^, peroxynitrite; SOD, superoxide dismutase; NO, nitric oxide; Fyn, Fyn kinase; GSSG, oxidized glutathione; GSH, reduced glutathione; DTT, dithiothreitol; DTNB, 5,5′-Dithiobis(2-nitrobenzoic acid).

As summarized in Table 2, both positive and negative findings are documented for the role of VGCC in the pathogenesis of CH-induced PH. This discrepancy may be due to differences in animal species/strains and hypoxic protocols employed. Even though the role of VGCC in CH-induced PH is controversial, redox regulation of VGCC is possible. L-type VGCC can be inhibited by NO but stimulated by ONOO^−^ [210], a product from NO and O_2_^.−^. S-nitrosothiols are NO-donors that can cause either L-type VGCC inhibition [211] or activation [210]. Application of H_2_O_2_ [212,213,214] and oxidized glutathione [71,72] leads to Ca^2+^ influx through L-type VGCC [212,213,214]. Further study showed that of the Cav1.2 subunit of L-type VGCC is glutathionylated by H_2_O_2_ and oxidized glutathione (GSSG), which increases channel open probability and inward Ca^2+^ currents [71,72] (Figure 5). The well-known vasoconstrictor ET-1 can also stimulate L-type VGCC-mediated Ca^2+^ increases in PASMCs from CH Wistar rats [180,215]. Interestingly, this response is plasma membrane depolarization independent [180] but PKC and Rho kinase-dependent [215]. Redox modulation in this process is possible as both PKC [216] and Rho kinase [25] can be activated by oxidation. This possibility is further supported by the fact that ET-1 increases ROS production in PASMCs [22,217,218]. Even though this hypothesis is not tested in pulmonary circulation, stimulation of L-type VGCC by ET-1 in isolated cardiac myocytes is demonstrated to be O_2_^.−^-mediated [219]. 

##### Transient Receptor Potential Canonical 1 and 6 (TRPC 1 and 6) Channels

TRPC1 and TRPC6 contribute to increased [Ca^2+^]_i_ in PASMCs as well as vasoconstriction following CH via involvement of SOCE and ROCE as indicated in Table 2. Their activities can be modulated by ROS. TRPC1 is mainly involved in SOCE in PASMC [178,200]. Administration of H_2_O_2_ increases STIM1/TRPC1 interactions and SOCE in cultured rat PASMCs [200]. TRPC6 is gated by diacylglycerol (DAG) [220,221,222,223]. The production of DAG can be facilitated by ROS. It is documented that NOX2-derived O_2_^.−^ during ischemia-reperfusion and exogenous H_2_O_2_ phosphorylate and activate phospholipase C (PLC) [222] that generates DAG from cleavage of membrane PIP_2_. More direct evidence for ROS modulation of TRPC6 is that H_2_O_2_ triggers TRPC6-dependent Ca^2+^ influx in aortic vascular smooth muscle cells, as well as contraction in endothelium-denuded aorta [224] (Figure 5). 

##### Transient Receptor Potential Vanilloid 4 (TRPV4) Channels

TRPV4, a member of the TRP channel superfamily, is a Ca^2+^ permeable non-selective cation channel. TRPV4 is expressed in all three layers of PAs, namely intimal (PAECs) [225,226,227], medial (PASMCs) [204,205] and adventitial (fibroblasts) layers [228]. Whereas TRPV4 expression in adventitial fibroblasts is upregulated by CH and contributes to excessive adventitial remodeling during the pathogenesis of PH [228], PASMC TRPV4 channels contribute to enhanced pulmonary vasoconstrictor reactivity following CH [204,205,206] (Table 2 for details). 

In contrast, Ca^2+^ influx conducted by endothelial TRPV4 is coupled to vasodilation. For example, Ca^2+^ sparklets via endothelial TRPV4 activate eNOS to cause PA vasodilation [225,226,227]. Moreover, the PA vasodilator effect of a TRPV4 agonist is absent when NOS is inhibited [226,227,229], supporting the possibility that eNOS is downstream of TRPV4 in PAECs. Another possible mechanism underlying TRPV4-mediated PA dilation is through small/intermediate conductance Ca^2+^-activated K^+^ channels (SK_Ca_/IK_Ca_)-dependent endothelium-derived hyperpolarizing factor (EDHF) responses [226]. Intravenous injection of the TRPV4 agonist GSK101790A lowers pulmonary arterial pressure in normal rats [229], although its therapeutic potential in PH has not been documented. 

Interestingly, TRPV4 activity in PAECs is enhanced by ROS. Extracellular H_2_O_2_ increases Ca^2+^ influx via TRPV4 in PAECs from mice and humans [230]. Mechanistically, this response requires TRPV4 phosphorylation by Fyn of the Src family kinases [230]. This process is facilitated by the scaffolding molecule CD36 that brings Fyn and TRPV4 together for efficient phosphorylation [231]. The possible phosphorylation site of TRPV4 that mediates its activation by H_2_O_2_ is serine 824 residue as demonstrated in human coronary artery endothelial cells channels [232]. Moreover, increased basal Ca^2+^ levels in PAECs from PAH rats are normalized by the SOD memetic TEMPOL, by mitochondria-targeted antioxidant MitoQ and by TRPV4 inhibitors [113], suggesting TRPV4 opening is maintained by endogenous ROS from mitochondria. This enhanced TRPV4-mediated Ca^2+^ entry in PAH animals contributes to proliferation and migration of PAECs [113]. Additionally, MitoQ attenuates TRPV4 agonist (GSK1016790A)-triggered Ca^2+^ influx observed in PAECs isolated from PAH rats [233] (Figure 5). Whether a similar ROS-induced TRPV4 activation mechanism in PASMCs contributes to elevated [Ca^2+^]_i_ and vasoconstriction in PH [204,205,206], however, remains to be determined.

##### Acid-Sensing Ion Channel 1 (ASIC1)

ASICs are members of degenerin/epithelial sodium channels that are activated by extracellular protons. There are at least six known different ASIC subunits (ASIC1a, ASIC1b, ASIC2a, ASIC2b, ASIC3, and ASIC4) that exist in mammals and are encoded by 4 genes (*ASIC1-4*). Some ASICs, such as ASIC1a homomeric channels and ASIC1a/2b heteromeric channels, also have the ability to conduct Ca^2+^, therefore directly participating in intracellular Ca^2+^ homeostasis regulation [234,235]. ASIC subunits are cysteine-rich and modified by the cellular redox status. Reducing agents potentiate ASIC1 activity while oxidizing agents decrease ASIC1 current [74,236,237,238,239]. In addition, oxidizing agents like H_2_O_2_ introduce intersubunit disulfide bonds, thereby decreasing the amount of ASIC1a present on the cell surface and reduce acid-evoked currents [74]. Consistent with these studies, we found that H_2_O_2_ inhibited, and PEG-catalase augmented ASIC1-dependent Ca^2+^ influx in PASMCs [28]. Using a Wistar rat model of hypobaric hypoxia-induced PH, we found that PASMC O_2_^.−^ levels are increased and H_2_O_2_ levels are decreased as a result of decreased SOD1 expression and activity [28]. This loss of endogenous H_2_O_2_ following CH contributes to the augmented ASIC1-dependent Ca^2+^ influx (Figure 5). The contribution of ASIC1 channels to CH-induced PH is summarized in Table 2.

##### Orai/STIM

STIM locates on SR. Translocation of STIM to plasma membrane upon SR depletion triggers SOCE via Orai channels. Orai/STIM participate in increases in resting cytosolic Ca^2+^ as well as SOCE following CH in PASMCs (Table 2). The resulting increase in intracellular Ca^2+^ levels is believed to couple to vasoconstriction but direct evidence for the contribution of Orai/STIM to PA constriction regulation is still absent. Orai/STIM-dependent SOCE can be regulated by ROS since oxidative stress upregulates STIM1 and Orai1 [200], increases STIM1/Orai1 interactions [200], facilitates STIM1 translocation to plasma membrane [73,240] and causes S-glutathionylation of cysteine 56 in STIM1 to trigger sustained Ca^2+^ entry that is independent of SR depletion [73] (Figure 5). 

##### Mechanosensitive Channels (Aka Stretch-Activated Channels) 

MSCs activated by plasma membrane stretch are permeable to Ca^2+^, therefore increasing [Ca^2+^]_i_. Ducret et al. [176] reported that MSC activity in PASMC is increased by CH exposure and contributes to myogenic tone of pulmonary arteries from CH rats whereas the PAs from normoxic animals do not exhibit tone (Table 2). Both O_2_^.−^ and ONOO^−^ are shown to facilitate stretch-included activation of MSCs [241] (Figure 5). 

#### 3.1.2. K^+^ Efflux

K^+^ channels selectively conduct outward K^+^ currents that are important in maintaining physiological membrane potential. K^+^ channels can be classified into different categories depending on the gating mechanisms. Within the pulmonary vasculature, four different types of K^+^ channels have been identified, including voltage-gated K^+^ channels (K_V_), Ca^2+^-activated K^+^ channels (K_Ca_), inwardly rectifying ATP-sensitive K^+^ channels (K_ATP_) and four transmembrane segments-2 pore K^+^ channels (K_2P_) [242,243,244]. Loss of outward K^+^ currents can lead to membrane depolarization. Since plasma membrane depolarization is observed in PASMCs after CH exposure [186,187], a role for suppression of K^+^ channels in this response has been investigated. Membrane depolarization is an important PA constriction stimulus because it activates Ca^2+^ channels such as VGCCs. Evidence for the involvement of different K^+^ channels will be discussed below. 

Loss of K_V_ channel function is coupled to enhanced L-type VGCC activity and PA constriction. This is evident by data indicating that general K_V_ channel blocker, 4-aminopyridine (4-AP), leads to L-type VGCC dependent increases in cytosolic Ca^2+^ as well as dose-dependent increases in basal pulmonary arterial tone [245]. Similar findings have been reported using blockers specific for K_V_7 (linopirdine and XE991) [246]. Therefore, K_V_ channel downregulation and reduced K_V_ currents observed in pulmonary vasculature [247,248,249,250,251,252,253] following CH are thought to mediate augmentation of PA constriction. This alteration is pathologically significant as restoration strategies have been shown to be beneficial in limiting PH [251,252,253]. K_V_1.5 and K_V_2.1 are of most importance in the context of CH-induced PH [247,248,249,250,251,252,253]. Detailed information is summarized in Table 3.

K_V_ channels are redox-sensitive primary via the modification of cysteine residues. NOX4 is reported to be colocalized with K_V_1.5 in PASMCs and oxidizes cysteine residues in K_V_1.5 [249]. NOX4 inhibition alleviates the CH-induced reduction in K_V_ currents [249]. Studies from Svoboda et al. [78] showed that ROS target the thiol (-SH) group of a cysteine (C581) residue at the C terminus of K_V_1.5, creating a sulfenic acid modification to K_V_1.5. Such modification results in a reduction of K_V_1.5 function by facilitating K_V_1.5 sequestration [78]. However, ROS modulation of K_V_2.1 within pulmonary circulation is understudied. In the central nervous system, ROS inhibit K_V_2.1 function by increasing its oligomerization [76,254]. Studies focused on molecular mechanisms of ROS-induced K_V_2.1 oligomerization revealed that K_V_2.1 oligomers are stabilized by disulfide bridges formed by oxidized cysteine residues at position 73 [75,76] and 710 [76].

**Table 3:** Suppression of K_V_ channels following CH exposure contributes to PH.

Experimental Model	CH Protocol	Down-Regulation	Functional Outcomes	Ref.
Primary PASMCs from normal rats, unspecified strain	Hypoxia (3% O_2_, 5% CO_2_, and 92% N_2_), Normoxia (5% CO_2_ in air), 48–72 h	K_V_1.2, K_V_1.5	N/A	[247]
Male Wistar rats	Hypoxia (10±0.5 % O_2_), Normoxia (room air), 21 days	K_V_1.1, K_V_1.5, K_V_1.6, K_V_2.1, and K_V_4.3	N/A	[248]
Primary PASMCs from male SD rats	Hypoxia (1% O_2_, 5% CO_2_, balance N_2_), Normoxia (5% CO_2_ in air), 48–72 h	K_V_1.5 and K_V_2.1	Decreased K_V_ currents	[249]
Primary PASMCs from normal SD rats	Hypoxia (3% O_2_, 5% CO_2_, and 92% N_2_), Normoxia (5% CO_2_ in air), 60–72 h	K_V_1.1, K_V_1.5, K_V_2.1, K_V_4.3, K_V_9.3	Loss of channels causes decreased K_V_ currents, membrane depolarization, increase cytosolic Ca^2+^	[250]
Male Wistar rats	Hypoxia (10% O_2_, balance N_2_), Normoxia (air), 21 days	K_V_1.5 and K_V_2.1 in PAs	Expression restoration prevents elevation in mPAP, RV hypertrophy	[251]
Male SD rats	Hypoxia (10 % O_2_), Normoxia (room air), 14–21 days	K_V_1.5 and K_V_2.1 in PAs	Expression restoration reverses and prevents PH indices following CH, including mPAP, PVR, RV hypertrophy, PA remodeling	[252]
Male SD rats	Hypoxia (380 mmHg), Normoxia (718 mmHg), 28 days	K_V_1.5 and K_V_2.1 in PAs	Expression restoration (1) rescues CH-induced suppression of K_V_ currents in PASMCs and (2) prevents RVSP elevation, RV hypertrophy and PA remodeling following CH	[253]
Female wild-type control mice (C57BL/6XCBA strain)	Hypoxia (10% O_2_), Normoxia (air), 14 days	N/A	K_V_7 activator dilates pre-constricted PAs and prevents PH indices following CH, including mRVP, RV hypertrophy and PA remodeling	[255]

Other K^+^ channels in pulmonary circulation, including K_ATP_, K_Ca_ and K_2P_ channels, are less well-studied in PH. Generally, these K^+^ channels exert a vasodilator effect in PAs when involved. The relevant findings are summarized in the following Table 4. Redox regulation of these K^+^ channels is possible but remains unclear in the pulmonary vasculature. As shown in Table 5, both activation and inhibition by ROS were observed in studies from other vascular beds.

### 3.2 ROS Participate in Ca^2+^ Sensitization 

#### 3.2.1 Rho Kinase Mediates Enhanced Ca^2+^ Sensitization 

**Figure 6:**
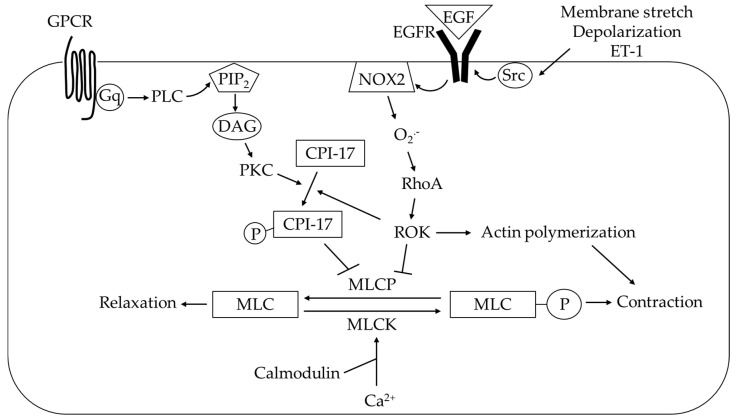
Summary of Ca^2+^ sensitization in pulmonary arterial smooth muscle cells. Myofilament Ca^2+^ sensitization is facilitated by ROS following CH. In particular, membrane stretch and endothelin 1 (ET-1) activate Src kinase-epidermal growth factor receptor (EGFR)-NADPH oxidase 2 (NOX2) signaling axis that contributes to CH-induced augmentation of Ca^2+^-independent pulmonary vasoconstriction and pulmonary hypertension. See text for details. GPCR, G protein-coupled receptor; PLC, phospholipase C; PIP_2_, phosphatidylinositol 4,5-bisphosphate; DAG, diacylglycerol; PKC, protein kinase C; O_2_^.−^, superoxide; ROK, Rho kinase; MLCP, myosin light chain phosphatase; MLCK, myosin light chain kinase; MLC, myosin light chain.

Enhanced vasoconstrictor responses in small PAs following CH can also be mediated by a Ca^2+^-independent mechanism in which vasoconstriction is independent of changes in intracellular Ca^2+^ levels. The contractile state of PASMCs results from the balance of MLCK and MLCP activities. Ca^2+^-independent vasoconstriction happens when phosphorylation of myosin light chain is maintained due to loss of MLCP activity. Phosphorylation of MLCP inhibits its function, which can be achieved by Rho kinase (ROK) either directly or indirectly via phosphorylated myosin light chain phosphatase inhibitor protein CPI-17 [281,282,283] (Figure 6). ROK is activated by GTP-bound RhoA [281,284]. Inhibition of ROK exerts a vasodilator effect on pulmonary vasculature [181,282,285] and therapeutic strategies targeting RhoA/ROK signaling is protective against CH-induced PH development in various animal models [282,286,287,288,289]. These observations suggest that RhoA/ROK represents a crucial mechanism underlying the pathogenesis of PH following CH. Pathophysiologically, ROK mediates the development of myogenic tone [19,21,181], along with enhanced vasoconstrictor reactivity to ET-1 [12,182] and membrane depolarizing stimuli following CH [18,19,20]. ROK can promote actin polymerization in PASMCs [19,288,290]. Our laboratory has demonstrated that such cytoskeletal remodeling actions of RhoA/ROK account for augmented PA constriction following CH [19]. ROK also facilities vasoconstriction by reducing eNOS expression as the inhibition of ROK increases eNOS expression in lungs of CH mice [282].

#### 3.2.2 ROS Regulation of RhoA/ROK Signaling 

Generally, the RhoA/ROK pathway can be activated by hypoxia [290], by plasma membrane depolarization [20,291,292,293,294] and by signals from G protein-coupled receptors, receptor tyrosine kinases, cytokine receptors and integrins [281,284]. Activated ROK phosphorylates the myosin phosphatase target subunit 1 (MYPT1) of MLCP at multiple threonine and serine residues [295], therefore inhibiting MLCP. Direct evidence for redox regulation of ROK activity is that the O_2_^.−^ donor, LY83583, increases ROK-dependent MYPT1 phosphorylation in PAs [27]. Additionally, ROS can mediate ROK activation in PAs in response to stimuli such as U46619 [25] and CH [12,20]. ROS are important in linking various pathogenic stimuli, such as receptor activation and membrane depolarization, to ROK-dependent Ca^2+^ sensitization in PASMCs in the setting of CH, therefore contributing to enhanced vasoconstriction [9]. 

The molecular mechanism by which ROS modulate RhoA/ROK signaling is not fully understood. Upregulation of RhoA under hypoxia appears to be downstream of ROS production [296]. RhoA is a member of Rho GTPase family whose function is regulated by guanine nucleotide-binding state [297]. RhoA is activated when binds to GTP with facilitation from guanine nucleotide exchange factors (GEFs) and deactivated by hydrolysis of GTP [297]. ROS have been shown to activate RhoA by targeting its redox-sensitive GXXXXGK(S/T)C motif, which determines guanine nucleotide dissociation [77,298]. Within this motif, cysteine residues 16 and 20 are critical for ROS modulation of RhoA activity [77,299]. 

Previous work from our laboratory has focused on delineating the contribution of ROS-dependent myofilament Ca^2+^ sensitization to vasoreactivity following CH [12,18,19,20,21,22]. PAs from CH rats have greater tone compared to vessels from control animals without a difference in [Ca^2+^]_i_ in PASMCs [19,21], suggesting the importance of the Ca^2+^ sensitization mechanism in maintaining elevated basal contractile state of PAs following CH. CH exposure also augments vasoconstriction to agonists independent of changes in Ca^2+^ because ET-1 [12] and membrane depolarization (KCl) [20] trigger greater constriction in Ca^2+^-permeabilized PAs from CH rats versus normoxic controls. Such differences are abolished by ROS scavengers alone [12,20], ROK inhibition alone [12,20] or combination of ROS scavenger and ROK [20]. Considering RhoA activation upon ET-1 [12] and KCl [20] stimulation in PAs requires ROS, it suggests ROS signal through ROK to facilitate CH-induced augmentation of myofilament Ca^2+^ sensitization. Furthermore, elevated KCl-induced vasoconstriction in Ca^2+^-clamped PAs from CH rats is normalized by NOX2 inhibition [18], indicating NOX2 as the enzymatic source of ROS involved in Ca^2+^ sensitization regulation. Upstream of NOX2 is epidermal growth factor receptor (EGFR) activation as EGFR is activated by KCl and contributes to KCl-induced ROS generation from NOX2 [18]. In summary, an EGFR-NOX2-ROK-mediated Ca^2+^ sensitization mechanism mediates CH-induced augmentation of PA vasoconstriction (Figure 6). This signaling is pathologically important because it participates in the development of PH following CH [22]. 

## 4. Conclusion/Perspective 

This review summarizes our understanding of ROS in enhanced PA constriction in the disease of PH, with an emphasis on CH-associated PH. Pathological ROS signaling following CH is the outcome of increased production from various enzymatic sources, as well as dysfunctional scavenging systems. ROS participate in PA vasomotor tone regulation following CH by modulating Ca^2+^ influx, K^+^ efflux and myofilament Ca^2+^ sensitization. Although ROS have convincingly reported to have an effect on these processes, little is known about the precise ROS-induced modifications to the relevant ion channels and signaling molecules. Limited studies indicate that ROS cause protein phosphorylation at serine residues and target cysteine residues to introduce modifications such as S-glutathionylation, disulfide formation and sulfenic acid formation. However, there are some potential pitfalls associated with such studies. First, the majority of current knowledge about ROS regulation of ion channels is gathered from experiments applying exogenous/extracellular oxidizing reagents, which fail to fully reflect the actions of intracellular ROS seen in PH. Second, there is a lack of pulmonary circulation-specific evidence regarding redox modifications. Third, it remains unclear how these identified ROS-induced modifications alter functions of the affected ion channels and signal transducers. Future studies are therefore needed to address these limitations. Furthermore, the upstream regulatory mechanisms that regulate enzymatic sources of ROS in PH are not fully understood. Such knowledge will be valuable in designing therapies specific to the disease while preserving the physiological functions of ROS. 

Considering the importance of ROS in mediating increases in pulmonary vascular resistance (PVR), a number of groups have attempted to develop novel therapeutic strategies for PH/PAH by preventing ROS production or scavenging ROS. Pre-clinical studies in PH/PAH animal models have shown promising results [3,29,30,31,32,33,34,35,36,37,300,301]. It is likely that oxidative signaling is also involved in human PH/PAH as oxidative stress is increased in chronic high-altitude residents [302] and in PH patients [303]. Moreover, in PAH patients, oxidative stress markers correlate with an adverse prognosis [304], and reducing oxidative stress by epoprostenol [305] or by recombinant human angiotensin converting enzyme type 2 (rhACE2) [306] is associated with decreases in PVR in small scale clinical trials. Aside from PVR, preserving or improving right heart function is a crucial goal for disease management [307] because cor pulmonale occurring during PH is fatal. Unfortunately, antioxidant therapy has shown little success in cardioprotection to date. Oral administration of antioxidant coenzyme Q (CoQ) improves left and right heart functions in PAH patients evaluated by echocardiography [308]. Additionally, cardiac magnetic resonance imaging demonstrates that the XO inhibitor, allopurinol, alleviates right ventricular hypertrophy in COPD-associated PH patients with severe airflow limitation [139]. However, in both trials, standard clinical cardiac function biomarkers, such as 6-minite walk distance and brain natriuretic peptide (BNP) levels, are not affected [139,308].

To our knowledge, these are the only available clinical trials [139,305,306,308] aimed at addressing the therapeutic potential of antioxidation strategies in PH/PAH to now. It is important to note that epoprostenol [305] and rhACE2 [306] used in clinical trials do not act primarily as antioxidants, and other classical antioxidants including SOD memetic and SOD/catalase mimetic have not been studied yet. Also, these pilot clinical trials have relatively small cohorts and fail to provide clinical details about optimal dose, treatment protocol, side effects and population generalizability. Therefore, multicenter double-blinded randomized controlled clinical trials are needed before drawing a firm conclusion about the therapeutic value of antioxidants in pulmonary hypertension.

## Figures and Tables

**Table 1:** Expression of NOX isoforms in the pulmonary vasculature.

NOX Isoform	Expression in PAEC	Expression in PASMC	ROS Generated
NOX1	Human [81,82,83], rat [84]	Human [85], rat [86,87], mouse [85,88]	O_2_^.−^ [83,85,86,87,88]
NOX2	Human [81,82,89,90], rat [84], mouse [89,91]	Rat [92]	O_2_^.−^ [81,89]
NOX3	Human [82]	N/A	O_2_^.−^ [93]
NOX4	Human [81,82,94], rat [84], mouse [95]	Human [90,96], rat [86,92], mouse [90]	O_2_^.−^ [82,97], H_2_O_2_ [82,95,96]

**Table 2:** Ca^2+^ influx in CH-induced PH.

Channel/Molecule	Alteration by CH	Functions
L-type VGCC	Increased current density [195], channel upregulation (Cav1.2) [196]	Positive: Mediate CH-induced enhanced pulmonary vascular tone [195] and PA vasoconstriction to KCl [195,196] and to L-type VGCC activator [195]
		Negative: 1. Without effects on basal [Ca^2+^]_i_ in PASMCs or basal PA tension [180] 2. Responsible for 30–40% of basal [Ca^2+^]_i_ in cultured PASMCs but not affect basal PA tone [178] 3. Do not contribute to increase PA wall basal [Ca^2+^]_i_ or elevated PA constriction to UTP following CH [17] 4. Do not contribute to CH-induced augmentation of PA myogenic tone [21] 5. CH-induced PH is not acutely alleviated by L-type VGCC inhibition in SD rats [197] or COPD patients [198,199]
T-type VGCC	Channel upregulation (Cav3.2) [196]	Positive: Mediate CH-induced augmented PA constriction to K^+^ and U-46619 [196]
		Negative: 1. Do not contribute to increase PA wall basal [Ca^2+^]_i_ following CH [17] 2. Do not contribute to CH-induced augmentation of PA myogenic tone [21]
TRPC1	Channel upregulation [177,178,200]	CH-induced PH [201,202]; SOCE in PASMC [178,200]; CH-induced augmented basal tone and vasoconstriction to 5-HT [202]
TRPC6	Channel upregulation [177,178,203]	CH-induced PH [202,203]; ROCE in PASMC [178]; augmented SOCE in PASMCs following CH [203]; basal tone under normoxia [202]; CH-induced augmented vasoconstriction to 5-HT [202]
TRPV4	Channel upregulation in PASMCs [204,205], increased channel activities in PASMCs [204,205]	1. CH-induced PH development [204,206] 2. CH-induced enhanced myogenic tone [204] and augmented vasoconstriction to serotonin [206] and TRPV4 agonist [205] but not to U46619 [204], PE [206] or ET-1 [206] in endothelium-disrupted PAs 3. Ca^2+^-induced Ca^2+^ release in PASMCs [205]
ASIC1	Unaltered expression [207]	Contribute to augmented SOCE and SOCE-induced vasoconstriction in PAs following CH [17]; CH-induced PH [207]
Orai1	Upregulation [14,200,203,208]	CH-induced increases in basal Ca^2+^ [14] and SOCE [14,200] in PASMCs
Orai2	Upregulation [14,203,208]	CH-induced increases in basal Ca^2+^ and SOCE in PASMCs [14]
Orai3	Unaltered expression [14]	CH-induced increases in basal Ca^2+^ and SOCE in PASMCs [14]
STIM1	Upregulation [200,208], unaltered expression [14]	CH-induced increases in basal Ca^2+^ [14] and SOCE [14,200] in PASMCs
STIM2	Upregulation [203,208]	Enhanced SOCE in PASMCs from PH patients [209]
MSC	Increased channel activities [176]	CH-induced augmentation of PA myogenic tone [19,21,176]

**Table 4:** Other K^+^ channels involved in CH-induced PH or PAH.

Type	Channel	Function	Ref.
K_ir_	K_ATP_	Gain of function protects against CH-induced PH indices including mPAP, RV hypertrophy and PA remodeling	[256]
K_Ca_	Large conductance K_Ca_ (BK_Ca_)	Gain of function protects against monocrotaline-induced PH, reduces PDGF-induced PASMC proliferation	[257]
		Loss of function does not affect PH development following CH	[258]
K_2P_	TREK-1 (K_2P_2.1)	Gain of function leads to PAEC hyperpolarization and PA relaxation	[259]
K_2P_	TWIK-2 (K_2P_6.1)	Gain of function leads to PAEC hyperpolarization and PA relaxation	[259]
K_2P_	TWIK-2 (KCNK6)	Loss of function results in increased RVSP, PA thickening, greater PA vasoconstrictor to U46619	[260]
		Loss of function causes PASMC depolarization, enhanced [Ca^2+^]_i_ and PA constriction to U46619	[261]
K_2P_	TASK-1 (KCNK3)	Loss of function favors proliferation of (PAEC, PASMC and fibroblast) and enhanced basal tone	[262]
		Gain of function protects against monocrotaline-induced PH	[262]
		Loss of function is without effects on CH-induced PH	[263]

**Table 5:** ROS modulation of K_ATP_ and K_Ca_ channels in cardiovascular system.

Outcome	ROS		
	O_2_^.−^	H_2_O_2_	ONOO^−^
K_ATP_ activation	Mesenteric artery SMC [264]	Mesenteric arteries [265] Cerebral arteries [266] Retinal microvessels [267] Cardiomyocytes [268]	Cerebral arteries [266] Internal carotid arteries [269]
K_ATP_ inhibition	Cerebral arteries [270]	A10 cell line [271]	N/A
K_Ca_ activation	Cerebral arteries [266]	Coronary arteries [272,273,274] Cerebral arteries [266,275]	Arteriolar SMC [276]
K_Ca_ inhibition	Coronary arteries [277] Cerebral arteries [270]	Renal arteries [278]	Coronary artery SMC [279] Gracilis arteries [280]

## Abbreviations

[Ca^2+^]_i_	Intracellular Ca^2+^ level
5-HT	Serotonin
ASIC	Acid-sensing ion channel
BH2	Dihydrobiopterin
BH4	Tetrahydrobiopterin
BK_Ca_	Large conductance Ca^2+^-activated K^+^ channels
BNP	Brain natriuretic peptide
Ca^2+^	Calcium
CH	Chronic hypoxia
COPD	Chronic obstructive pulmonary disease
CoQ	Coenzyme Q
DAG	Diacylglycerol
DTNB	5,5′-Dithiobis(2-nitrobenzoic acid)
DTT	Dithiothreitol
EC	Endothelial cell
EGFR	Epidermal growth factor receptor
eNOS	Endothelial nitric oxide synthase
ET-1	Endothelin-1
ETC	Electron transport chain
FAD	Flavin adenine dinucleotide
GPCR	G protein coupled receptor
GSH	Reduced glutathione
GSSG	Oxidized glutathione
H_2_O_2_	Hydrogen peroxide
HPV	Hypoxic pulmonary vasoconstriction
IK_Ca_	Intermediate conductance Ca^2+^-activated K^+^ channels
IP_3_	Inositol triphosphate
K^+^	Potassium
K_2P_	Four transmembrane segments-2 pores K^+^ channels
K_ATP_	ATP-sensitive K^+^ channels
K_Ca_	Ca^2+^-activated K^+^ channels
KO	Knockout
K_V_	Voltage-gated K^+^ channels
MAO	Monoamine oxidase
MitoROS	Mitochondria-derived ROS
MLCK	Myosin light chain kinase
MLCP	Myosin light chain phosphatase
mPAP	Mean pulmonary arterial pressure
MSC	Mechanosensitive channel
MYPT1	Myosin phosphatase target subunit 1
Na^+^	Sodium
NADPH	Reduced nicotinamide adenine dinucleotide phosphate
NO	Nitric oxide
NOX	NADPH oxidase
O_2_^.−^	Superoxide anion
ONOO^−^	Peroxynitrite ion
PA	Pulmonary artery
PAEC	Pulmonary arterial endothelial cell
PAH	Pulmonary arterial hypertension
PASMC	Pulmonary arterial smooth muscle cell
PEG-SOD	Polyethylene glycol-conjugated SOD
PH	Pulmonary hypertension
PIP_2_	Phosphatidylinositol 4,5-bisphosphate
PLC	Phospholipase C
PVR	Pulmonary vascular resistance
rhACE2	Recombinant human angiotensin converting enzyme type 2
ROC	Receptor-operated channel
ROCE	Receptor-operated Ca^2+^ entry
ROK	Rho kinase
ROS	Reactive oxygen species
RV	Right ventricular
RVSP	Right ventricular systolic pressure
SK_Ca_	Small conductance Ca^2+^-activated K^+^ channels
SMC	Smooth muscle cell
SOC	Store-operated channel
SOCE	Store-operated Ca^2+^ entry
SOD	Superoxide dismutase
SR	Sarcoplasmic reticulum
STIM	Stromal interaction molecule
TRPC	Transient receptor potential canonical
TRPV4	Transient receptor potential vanilloid 4
VGCC	Voltage-gated calcium channel
WHO	World health organization
WT	Wild-type
XDH	Xanthine dehydrogenase
XO	Xanthine oxidase
XOR	Xanthine oxidoreductase
